# Associations of Self-Reported Physical Activity with Gut Microbial Diversity and Community Structure in Young, Apparently Healthy Korean Men

**DOI:** 10.3390/sports14100436

**Published:** 2026-10-07

**Authors:** Byunghun So, Junho Jang, Jinhan Park, Chounghun Kang

**Affiliations:** 1 Department of Health and Exercise Science, College of Graduate School, Inha University, Incheon 22212, Republic of Korea; sportshun@gmail.com (B.S.); jangju2489@gmail.com (J.J.); sportsjinhan@gmail.com (J.P.); 2 Department of Physical Education, College of Education, Inha University, Incheon 22212, Republic of Korea

**Keywords:** physical activity (PA), gut microbiota, alpha diversity, beta diversity, metabolic equivalent (MET)

## Abstract

Evidence linking physical activity (PA) to the gut microbiota in Korean populations remains limited. We examined associations of self-reported PA with gut microbial diversity and community structure in 49 young, apparently healthy Korean men. Participants were classified into study-specific categories based on total metabolic equivalent minutes per week (MET-min/week): C1 (≤1500), C2 (1501–3000), and C3 (≥3001). Fecal microbiota were profiled using 16S rRNA gene V4 sequencing, and diversity analyses used 100 repeated rarefactions to 22,000 reads per sample. In primary continuous analyses, total MET-min/week was positively correlated with the Shannon index (ρ = 0.3567, q = 0.0356) after false discovery rate (FDR) correction, whereas associations with Observed features and Fisher’s alpha did not remain significant. Total MET-min/week was also associated with variation in microbial community composition across all four beta-diversity metrics, explaining 2.7–3.3% of the variation. In secondary categorical analyses, alpha diversity did not differ significantly among PA categories after FDR correction, whereas Jaccard and unweighted UniFrac showed FDR-significant differences. No robust genus-level association was identified after FDR correction and compositional sensitivity analysis. These exploratory findings indicate small cross-sectional associations and should be interpreted cautiously because the primary analyses were largely unadjusted for potential lifestyle confounders.

## 1. Introduction

Physical activity (PA) refers to any movement produced by skeletal muscles that results in energy expenditure above resting levels. Regular PA contributes to physical fitness and is associated with a lower risk of metabolic and cardiovascular diseases, as well as improvements in brain health and overall well-being [1,2,3]. The World Health Organization (WHO) recommends that adults perform at least 150 min of moderate-intensity aerobic PA per week [4]. Nevertheless, approximately 31% of adults worldwide do not meet recommended PA levels [5], underscoring the continuing public health burden associated with physical inactivity [4,5].

Advances in next-generation sequencing (NGS) and metagenomic technologies have enabled detailed characterization of the human gut microbiota [6]. The gastrointestinal tract contains a dense and taxonomically diverse microbial community in which members of the Firmicutes and Bacteroidetes phyla are frequently predominant, although their relative abundances vary substantially among individuals [7,8,9]. The gut microbiota contributes to nutrient metabolism, intestinal epithelial homeostasis, immune regulation, and the production of microbial metabolites, including short-chain fatty acids such as acetate, propionate, and butyrate [10,11,12]. However, gut microbial composition is influenced by multiple host and environmental factors, including ethnicity [13], habitual diet [14,15], antibiotic exposure [16], and living environment [17,18].

PA has been proposed as a modifiable factor associated with gut microbial composition [12]. Observational and intervention studies have reported associations of higher PA or regular exercise with microbial diversity, community structure, and the relative abundance of taxa previously linked to short-chain fatty acid production or host energy metabolism [19,20,21]. However, findings have not been consistent across populations or study designs, and reported associations may differ according to participant characteristics, exercise intensity and duration, habitual diet, adiposity, and training status [22,23,24]. These biological and contextual sources of heterogeneity may be further compounded by methodological variation because uneven sequencing depth, bioinformatic feature-processing procedures, and the choice of diversity metric can influence the estimated diversity and apparent differences among microbial communities [25,26,27]. Consequently, the relationship of PA with gut microbial diversity and taxonomic composition remains incompletely understood and requires further investigation across different populations and analytical settings [3,28].

The generalizability of previous findings may also be limited by ethnicity, habitual dietary patterns, and environmental context [13,14,15]. Traditional Korean dietary practices, including the consumption of fermented foods such as kimchi, may represent population-specific factors influencing gut microbial composition [29,30,31,32]. Nevertheless, evidence examining the associations of self-reported PA with gut microbial diversity and community structure in Korean adults remains limited. Therefore, this exploratory study aimed to investigate the associations of self-reported PA with gut microbial alpha diversity, beta diversity, and genus-level taxonomic profiles in young, apparently healthy Korean men. The primary analyses examined total metabolic equivalent minutes per week (MET-min/week) as a continuous exposure in relation to alpha and beta diversity. Comparisons across three study-specific, data-derived PA categories and genus-level analyses were treated as secondary, exploratory analyses.

## 2. Materials and Methods

### 2.1. Participants

Forty-nine apparently healthy Korean men aged 20–29 years were included in the analysis. Participants were recruited primarily from a university student population. Each participant attended a single study visit at enrollment, during which a stool sample was collected, and an intake survey was completed. Health status was determined based on self-reported health information, disease history, and current/past medication history obtained during the intake survey. Participants were considered apparently healthy if they reported no history of chronic disease, no alcohol consumption for ≥3 days before stool collection, and no use of antibiotics or other medications known to affect the gut microbiota within the past month. No medical examination or laboratory screening was performed to define healthy status. A single dietary-pattern item, classified as meat-preferring or plant-preferring, was included in the intake survey. However, because this item did not quantify dietary intake and was not based on a validated dietary assessment instrument, dietary data were not included in the analysis. Other lifestyle, health-related, dietary, supplement-use, and training variables were not systematically assessed in a form suitable for covariate adjustment. All participants provided written informed consent, and the study was approved by the Inha University IRB (INHA-210517-7AR2).

### 2.2. Physical Activity Assessment (IPAQ-SF)

Physical activity was assessed with the International Physical Activity Questionnaire-Short Form (IPAQ-SF) [33]. Total MET-min/week was calculated from separately reported walking, moderate-intensity, and vigorous-intensity activities using the standard IPAQ MET coefficients of 3.3, 4.0, and 8.0, respectively. Before analysis, IPAQ-SF responses were screened for implausible activity durations. All reported walking, moderate-intensity, and vigorous-intensity activity durations were ≤180 min/day, requiring no truncation. Walking, moderate-intensity, vigorous-intensity, and total MET-min/week were summarized descriptively (Supplementary Table S1). An item intended to assess sitting behavior was included in the questionnaire. However, its response options recorded days per week rather than sitting duration. Therefore, a valid sitting-time variable could not be derived and was not analyzed. Intensity-specific PA components were summarized descriptively but were not modeled as separate microbiome exposures. Participants were classified into three study-specific categories based on self-reported total MET-min/week: C1 (≤1500 MET-min/week), C2 (1501–3000 MET-min/week), and C3 (≥3001 MET-min/week). The cut-points were selected after reviewing the distribution of IPAQ-SF total MET-min/week but before examining any microbiome outcomes. They were chosen pragmatically to create ordered lower-, intermediate-, and higher-activity strata while avoiding very small group sizes. These thresholds were not prospectively documented in the study protocol or in a formal statistical analysis plan and are described as study-specific, data-derived categories rather than prespecified categories. They do not correspond to the canonical IPAQ Low, Moderate, and High classifications, which also incorporate frequency-, duration-, and intensity-specific criteria. Accordingly, C1–C3 were treated as study-specific categories of self-reported IPAQ-SF total MET-min/week and were not intended to indicate objectively measured PA levels. These categories were used for secondary exploratory analyses, whereas continuous total MET-min/week was treated as the primary exposure.

### 2.3. Collection and DNA Extraction

Participants self-collected fecal samples using a standardized kit. Participants were instructed to collect stool samples when they were in their usual, good physical condition and to provide a sample collected within the previous 12 h. They were also instructed to freeze the sample at −20 °C as soon as possible after defecation. Samples were transported on ice and stored at −80 °C until analysis. No samples exceeded the requested 12 h collection window, and no repeated freeze–thaw cycles occurred before analysis. However, the time of day of stool collection, the exact interval between defecation and freezing, and acute strenuous exercise immediately before stool collection were not standardized or recorded. DNA was extracted from 200 mg of stool using the SPINeasy DNA Kit for Feces (MP Biomedicals, Irvine, CA, USA) and homogenized with a FastPrep-24^TM^ 5G bead homogenizer (MP Biomedicals, Irvine, CA, USA). DNA quality was verified by 1% agarose gel electrophoresis and ChemiDoc (Bio-Rad, Hercules, CA, USA) imaging.

### 2.4. 16S rRNA-Based Amplicon Sequencing

The V4 region of the 16S rRNA gene was amplified using primers containing locus-specific overhang sequences. The primer sequences, synthesized by Bioneer (Daejeon, Republic of Korea), were as follows: 515F (5′-TCGTCGGCAGCGTCAGATGTGTATAAGAGACAG-GTGCCAGCMGCCGCGGTAA-3′) and 806R (5′-GTCTCGTGGGCTCGGAGATGTGTATAAGAGACAG-GGACTACHVGGGTWTCTAAT-3′). Amplification was performed using a T100 Thermal Cycler (Bio-Rad, Hercules, CA, USA) with a reaction mix containing 5 ng of template DNA, 1 µL of Amplicon PCR Forward Primer, 1 µL of Amplicon PCR Reverse Primer, and 12 µL of 2× KAPA HiFi HotStart Ready Mix (KAPA Biosystems, InC., Wilmington, MA, USA). The thermal cycling conditions for the PCR included an initial denaturation step at 95 °C for 3 min, followed by 25 cycles of 95 °C for 30 s, 55 °C for 30 s, and 72 °C for 30 s, with a final extension at 72 °C for 5 min, after which the samples were held at 4 °C. Indexes and Illumina sequencing adapters were added using the Nextera XT Index Kit v2 Set A (Illumina, San Diego, CA, USA) via index-PCR. The index-PCR was conducted with the following cycling conditions: initial denaturation at 95 °C for 3 min, 8 cycles of 95 °C for 30 s, 55 °C for 30 s, and 72 °C for 30 s, followed by a final extension at 72 °C for 5 min. After PCR amplification, unincorporated primers and dimer species were removed using the AMPure XP Reagent (Beckman Coulter, Inc., Brea, CA, USA) for PCR clean-up. The library concentration was determined using a Qubit™ 4 Fluorometer (Thermo Fisher Scientific, Waltham, MA, USA) with the dsDNA HS Assay Kit. Sequencing was performed on an iSeq 100 Sequencing System using the iSeq 100 i1 Reagent v2 (300-cycle) in a 2 × 150-bp paired-end configuration, incorporating PhiX Control v3 for quality control.

### 2.5. Bioinformatic Processing

Raw reads were processed in QIIME 2 v2024.5 using the Divisive Amplicon Denoising Algorithm 2 (DADA2) denoise-single workflow for quality filtering, denoising, chimera removal, and inference of amplicon sequence variants (ASVs). Although sequencing was performed in a 2 × 150-bp paired-end configuration, only the forward reads were retained for downstream analysis. Forward- and reverse-read quality profiles were generally comparable over most read positions. To evaluate whether paired-end merging was feasible, an additional DADA2 denoise-paired quality-control analysis was performed using the full available forward and reverse read lengths and requiring a minimum overlap of 12 nt. Of 2,363,232 denoised read pairs, only 4464 (0.19%) successfully merged, corresponding to a merge failure rate of 99.81%. This indicated that the 2 × 150-bp configuration did not provide sufficient overlap for reliable reconstruction of the 515F/806R V4 amplicon. Accordingly, the final microbiome analysis was performed using forward reads only. Demultiplexing yielded 3,133,927 read pairs across the 49 samples, comprising 3,133,927 forward reads and an equal number of reverse reads. The 3,133,927 forward reads entered the DADA2 denoise-single workflow. DADA2 was run with trunc-len = 150, trim-left = 19, max-ee = 2.0, trunc-q = 2, and chimera-method = consensus. The first 19 nucleotides were removed from the 5′ end to exclude the residual 515F primer region. Of the 3,133,927 input forward reads, 2,639,368 passed quality filtering, 2,608,196 remained after denoising, and 2,549,665 were retained after chimera removal. A total of 58,531 reads, corresponding to 2.24% of the denoised reads, were removed as chimeric. DADA2 generated 3656 ASVs, and all 49 samples were retained. All representative ASV sequences were 131 bp in length. Taxonomic assignment was performed using the QIIME 2 feature-classifier classify-sklearn method with a confidence threshold of 0.7. A Naive Bayes classifier was trained using SILVA 138 SSURef NR99 reference sequences extracted for the 515F/806R V4 region. The reference sequences were truncated to 131 bp and oriented in the forward direction to match the representative ASV sequences used in this study. Features not assigned to the domain Bacteria and features classified as chloroplast or mitochondria were removed. The final bacterial feature table contained 3034 ASVs and 2,527,242 reads across all 49 samples. No additional minimum-count, prevalence, or low-variance filtering was applied to the bacterial feature table used for alpha- and beta-diversity analyses. A reference-based phylogenetic tree was generated using the QIIME 2 fragment-insertion sepp method with the SILVA 128 SEPP reference artifact, sepp-refs-silva-128.qza. All retained bacterial ASVs were successfully placed into the reference phylogeny. The resulting bacterial feature table and insertion tree were used for the alpha- and beta-diversity analyses. SILVA 138 was used for taxonomic classification, whereas the SILVA 128 SEPP reference resource was used specifically for phylogenetic fragment insertion.

### 2.6. Statistical Analysis

Raw total MET-min/week was treated as the primary continuous exposure. Analyses based on the three study-specific PA categories were considered secondary and exploratory. Log1p-transformed total MET-min/week was used as a sensitivity analysis for the continuous beta-diversity findings. Participant characteristics were compared using one-way analysis of variance. Sequencing-depth differences among PA categories were assessed using a Kruskal–Wallis test based on pre-rarefaction library size. Spearman rank correlations were used to examine associations of library size with total MET-min/week and the unrarefied observed ASV count. The bacterial ASV table was rarefied 100 times without replacement to 22,000 reads per sample. This depth retained all 49 participants because the minimum library size was 22,766 reads, and the alpha-rarefaction curves had approached a plateau at this depth. Observed features, the Shannon index, and Fisher’s alpha were calculated for each rarefied table. For each participant, the median of the 100 rarefaction-specific values was used as the summary value for each alpha-diversity metric. Primary associations between raw total MET-min/week and alpha diversity were assessed using two-sided Spearman rank correlations. Bias-corrected and accelerated (BCa) 95% bootstrap confidence intervals for Spearman’s ρ were estimated from 10,000 participant-level resamples. Partial Spearman rank correlations adjusted for age and BMI were performed as sensitivity analyses. Secondary comparisons among PA categories were assessed using Kruskal–Wallis tests followed by pairwise two-sided Wilcoxon rank-sum tests. Epsilon-squared was reported for the global Kruskal–Wallis tests. The continuous Spearman correlations and global Kruskal–Wallis tests were also repeated for each of the 100 rarefied tables to assess stability across repeated rarefactions. The Benjamini–Hochberg correction was applied across the three alpha-diversity metrics within each iteration.

Jaccard, Bray–Curtis, Jensen–Shannon, and unweighted UniFrac distance matrices were calculated separately for each of the 100 rarefied feature tables. Jensen–Shannon distance was calculated in QIIME 2 v2024.5 using the q2-diversity beta action with the jensenshannon metric and represents the square root of the Jensen–Shannon divergence. For each metric, the 100 rarefaction-specific distance matrices were averaged element-wise to obtain a single averaged distance matrix, which was used for the main PERMANOVA analyses and PCoA visualization.

Standardized raw total MET-min/week was entered as the primary explanatory variable in separate PERMANOVA models for the four averaged distance matrices. Sensitivity analyses were performed after adjustment for standardized age and BMI and using standardized log1p-transformed total MET-min/week. Secondary categorical analyses assessed global and pairwise differences among PA categories using PERMANOVA implemented with the adonis2 function in the vegan package v2.7-5 in R v4.5.2. Multivariate dispersion was evaluated using PERMDISP based on distances to group spatial medians. PERMANOVA and PERMDISP used 9999 permutations, and PERMANOVA effect sizes were reported as R^2^. Principal coordinates analysis was performed using the averaged distance matrix for each metric. The continuous PERMANOVA models and global categorical PERMANOVA and PERMDISP tests were also repeated using each of the 100 rarefaction-specific distance matrices. Rarefaction-specific PERMANOVA pseudo-F statistics, R^2^ values, and *p*-values were summarized. Within each iteration and model, Benjamini–Hochberg correction was applied across the four distance metrics. These repeated-rarefaction analyses were used to assess the stability of the findings across repeated subsampling of the same participants and were not treated as independent biological replications.

Genus-level analyses were exploratory and used the unrarefied bacterial feature and taxonomy tables. ASVs assigned to named genera were aggregated at the genus level. Relative abundances for the correlation analyses were calculated using the complete retained bacterial community within each participant as the denominator before genus-level filtering. Named genera detected in at least 10% of participants (≥5 of 49) were retained, resulting in 151 genera for hypothesis testing. Associations between raw total MET-min/week and genus-level relative abundances were assessed using two-sided Spearman rank correlations. ANCOM-BC2 v2.12.0 was used as a compositional sensitivity analysis with standardized total MET-min/week as the continuous predictor, a prevalence cutoff of 10%, Benjamini–Hochberg adjustment, and a pseudo-count sensitivity analysis. Only findings supported by the pseudo-count sensitivity criterion were considered robust.

Benjamini–Hochberg FDR correction was applied within defined families of related tests. The primary and age- and BMI-adjusted alpha-diversity analyses were corrected separately across the three alpha-diversity metrics. The raw-MET, age- and BMI-adjusted, and log1p-transformed beta-diversity models were corrected separately across the four distance metrics. Global categorical alpha-diversity, PERMANOVA, and PERMDISP analyses were treated as separate testing families. For pairwise analyses, correction was applied across the three group comparisons separately for each metric and test type. Genus-level Spearman correlations were corrected across all 151 tested genera. A two-sided *p* < 0.05 or FDR-adjusted q < 0.05 was considered statistically significant, as applicable. During the revision of this manuscript, ChatGPT (OpenAI; GPT-5 series, with model versions varying as the service was updated; accessed May–September 2026) was used to assist with the development and troubleshooting of R analysis scripts and to review the reporting and interpretation of statistical results. All statistical analyses were executed and verified by the authors, and all analytical decisions and interpretations were independently reviewed by the authors.

## 3. Results

### 3.1. Participant Characteristics

Forty-nine apparently healthy Korean men (age, 22.1 ± 2.1 y; height, 175.1 ± 5.2 cm; weight, 72.8 ± 8.8 kg; BMI, 23.7 ± 2.6 kg/m^2^) were stratified into three study-specific, data-derived PA categories based on total MET-min/week: C1 (≤1500 MET-min/week), C2 (1501–3000 MET-min/week), and C3 (≥3001 MET-min/week). Age, height, weight, and BMI did not differ significantly among the categories. Overall total MET-min/week was 2919.8 ± 1770.3, with a median of 2733 (1410–4266) and a range of 132–7026 MET-min/week. As expected from the category definitions, total MET-min/week differed significantly among the categories (*p* < 0.001). Distributions of walking, moderate-intensity, and vigorous-intensity PA are provided in Supplementary Table S1.

### 3.2. Alpha Diversity in Relation to Self-Reported PA

The bacterial feature table used for diversity analyses contained 3034 ASVs and 2,527,242 reads across all 49 samples, with a minimum library size of 22,766 reads. Pre-rarefaction library size did not differ significantly among PA categories (*p* = 0.2174) and was not significantly associated with total MET-min/week (Spearman’s ρ = 0.1882, *p* = 0.1952). However, library size was positively correlated with the unrarefied observed ASV count (ρ = 0.4823, *p* = 0.000449; Supplementary Table S2).

**Figure 1:**
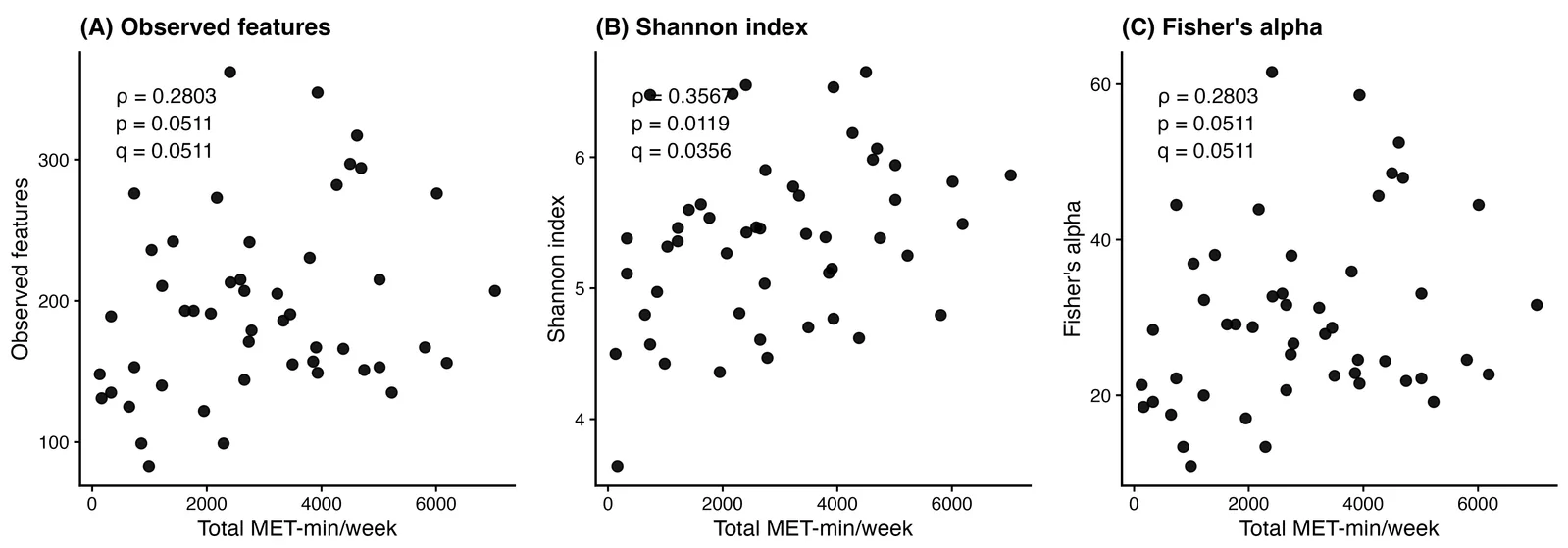
Associations between total MET-min/week and alpha diversity. Scatter plots show the relationships of raw total MET-min/week with (**A**) Observed features, (**B**) the Shannon index, and (**C**) Fisher’s alpha across all 49 participants. Each point represents the participant-level median calculated from 100 repeated rarefactions to 22,000 reads per sample. Statistical inference was based on two-sided Spearman rank correlations. Plot annotations show Spearman’s ρ, raw *p*-values, and Benjamini–Hochberg FDR-adjusted q-values. Corresponding statistical results, 95% bootstrap confidence intervals, and repeated rarefaction stability are provided in Supplementary Table S5.

In the primary continuous analyses, total MET-min/week was positively correlated with the Shannon index (Spearman’s ρ = 0.3567, 95% BCa bootstrap CI = 0.0821 to 0.5812, *p* = 0.0119, q = 0.0356). Associations with Observed features (ρ = 0.2803, *p* = 0.0511, q = 0.0511) and Fisher’s alpha (ρ = 0.2803, *p* = 0.0511, q = 0.0511) were not significant after FDR correction (Figure 1; Supplementary Table S5A). In the age- and BMI-adjusted sensitivity analyses, the association with the Shannon index remained significant (partial Spearman’s ρ = 0.3697, *p* = 0.0105, q = 0.0316). Associations with Observed features and Fisher’s alpha were also significant after FDR correction (both partial Spearman’s ρ = 0.2945, *p* = 0.0445, q = 0.0445; Supplementary Table S5B). Across the 100 rarefaction-specific analyses, the association with the Shannon index met the FDR-adjusted q < 0.05 threshold in all 100 iterations, whereas the associations with Observed features and Fisher’s alpha met this threshold in 29 of 100 iterations (Supplementary Table S5C).

**Figure 2:**
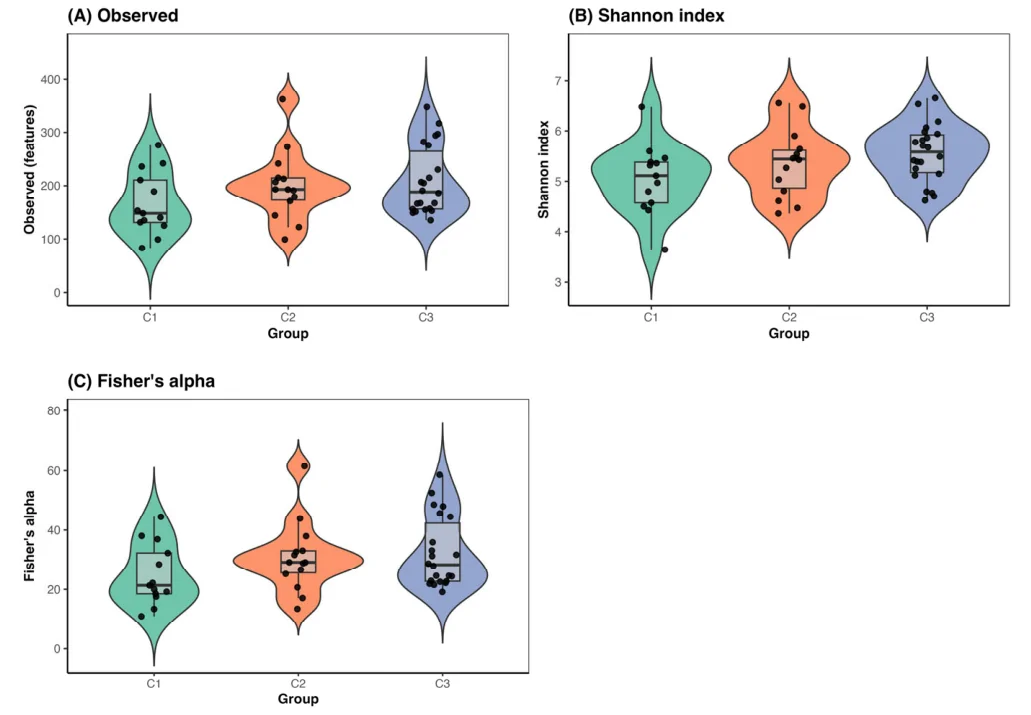
Alpha diversity across study-specific self-reported physical activity (PA) categories. PA categories were defined as C1 (≤1500 MET-min/week), C2 (1501–3000 MET-min/week), and C3 (≥3001 MET-min/week). For each participant, the plotted values represent the median of 100 repeated rarefaction values obtained at 22,000 reads per sample. Violin plots with embedded boxplots and individual data points show (**A**) Observed features, (**B**) the Shannon index, and (**C**) Fisher’s alpha. Boxplots indicate the median and interquartile range, and individual points represent participants. Group-wise summaries and global and pairwise statistical comparisons are provided in Supplementary Table S3 and Supplementary Table S4, respectively.

In the secondary exploratory categorical analyses, alpha-diversity values were numerically lower in C1 than in C2 and C3 across all three metrics (Figure 2; Supplementary Table S3). Global Kruskal–Wallis tests showed no significant differences among PA categories for Observed features (χ^2^ = 4.207, ε^2^ = 0.048, *p* = 0.1220, q = 0.1220), the Shannon index (χ^2^ = 5.588, ε^2^ = 0.078, *p* = 0.0612, q = 0.1220), or Fisher’s alpha (χ^2^ = 4.207, ε^2^ = 0.048, *p* = 0.1220, q = 0.1220). No pairwise comparison remained significant after FDR correction (Supplementary Table S4). None of the global PA-category tests met the FDR-adjusted q < 0.05 threshold across the 100 rarefaction-specific analyses (Supplementary Table S5C).

### 3.3. Beta Diversity in Relation to Self-Reported PA

Beta diversity was assessed using Jaccard, Bray–Curtis, Jensen–Shannon, and unweighted UniFrac distances. In the primary continuous analyses using the averaged distance matrices, standardized raw total MET-min/week was associated with variation in microbial community composition for Jaccard (pseudo-F = 1.405, R^2^ = 0.0290, *p* = 0.0080, q = 0.0320), Bray–Curtis (pseudo-F = 1.600, R^2^ = 0.0329, *p* = 0.0226, q = 0.0328), Jensen–Shannon (pseudo-F = 1.378, R^2^ = 0.0285, *p* = 0.0378, q = 0.0378), and unweighted UniFrac (pseudo-F = 1.315, R^2^ = 0.0272, *p* = 0.0246, q = 0.0328). The corresponding R^2^ values ranged from 0.0272 to 0.0329, indicating that total MET-min/week accounted for approximately 2.7–3.3% of the variation in community composition across the four metrics (Supplementary Table S8A).

In age- and BMI-adjusted sensitivity analyses, total MET-min/week was associated with community composition across all four metrics, with FDR-adjusted q = 0.0146 for each metric (Supplementary Table S8B). Log1p-transformed total MET-min/week was also associated with Jaccard (pseudo-F = 1.547, R^2^ = 0.0319, *p* = 0.0018, q = 0.0036), Bray–Curtis (pseudo-F = 1.941, R^2^ = 0.0397, *p* = 0.0017, q = 0.0036), Jensen–Shannon (pseudo-F = 1.677, R^2^ = 0.0345, *p* = 0.0032, q = 0.0043), and unweighted UniFrac (pseudo-F = 1.289, R^2^ = 0.0267, *p* = 0.0320, q = 0.0320) (Supplementary Table S8C). Across the 100 rarefaction-specific analyses, the raw and age- and BMI-adjusted models met the FDR-adjusted q < 0.05 threshold in all 100 iterations for all four metrics. The log1p-transformed models met this threshold in all 100 iterations for Jaccard, Bray–Curtis, and Jensen–Shannon and in 97 of 100 iterations for unweighted UniFrac (Supplementary Table S8D). Rarefaction-specific distributions of PERMANOVA pseudo-F statistics, R^2^ values, and *p*-values are provided in Supplementary Table S8E.

**Figure 3:**
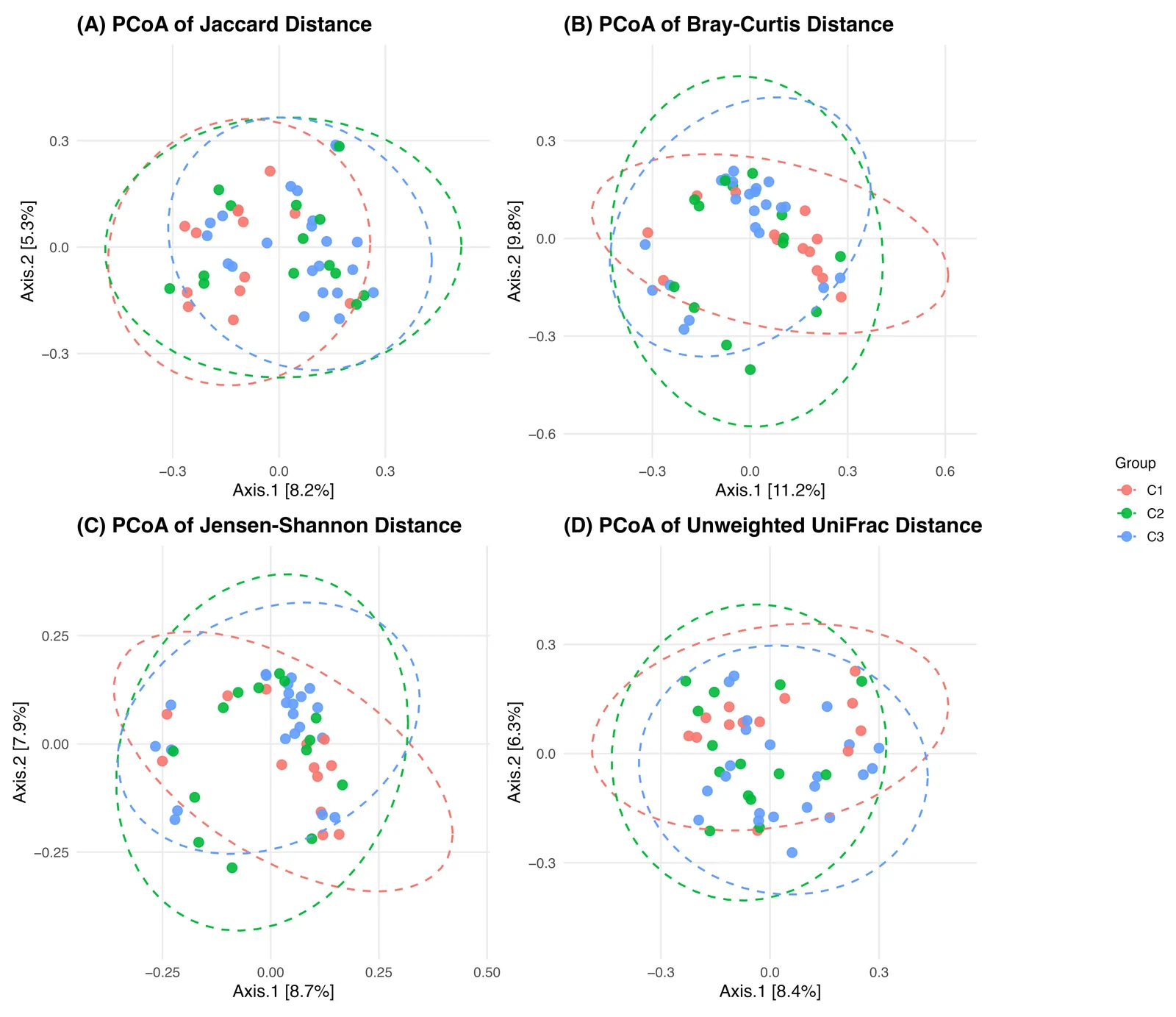
Beta diversity across study-specific self-reported physical activity (PA) categories. PA categories were defined as C1 (≤1500 MET-min/week), C2 (1501–3000 MET-min/week), and C3 (≥3001 MET-min/week). Principal coordinates analysis (PCoA) plots were generated from distance matrices averaged across 100 rarefactions to 22,000 reads per sample. Panels show (**A**) Jaccard, (**B**) Bray–Curtis, (**C**) Jensen–Shannon, and (**D**) unweighted UniFrac distances. Points represent individual samples, and dashed lines indicate 95% normal-theory ellipses for each PA category. Global categorical PERMANOVA, PERMDISP, and repeated-rarefaction stability results are provided in Supplementary Table S6, and pairwise results are provided in Supplementary Table S7.

In the secondary exploratory categorical analyses, global PERMANOVA showed differences among PA categories for Jaccard (pseudo-F = 1.221, R^2^ = 0.0504, *p* = 0.0191, q = 0.0432) and unweighted UniFrac (pseudo-F = 1.232, R^2^ = 0.0509, *p* = 0.0216, q = 0.0432). Bray–Curtis (pseudo-F = 1.179, R^2^ = 0.0488, *p* = 0.1471, q = 0.1783) and Jensen–Shannon (pseudo-F = 1.117, R^2^ = 0.0463, *p* = 0.1783, q = 0.1783) were not significant after FDR correction (Figure 3, Supplementary Table S6A). Exploratory pairwise PERMANOVA identified an FDR-significant C1–C3 difference only for Jaccard distance. Detailed pairwise results are provided in Supplementary Table S7A.

Global PERMDISP showed no significant differences in multivariate dispersion for any of the four metrics, and no pairwise PERMDISP comparison remained significant after FDR correction (Supplementary Tables S6B and S7B). Across the 100 rarefaction-specific categorical analyses, Jaccard and unweighted UniFrac yielded raw *p* < 0.05 in all 100 iterations and FDR-adjusted q < 0.05 in 71 of 100 iterations. Bray–Curtis and Jen-sen-Shannon did not yield raw *p* < 0.05 in any iteration. No global PERMDISP result met the FDR-adjusted q < 0.05 threshold across the 100 repeated rarefactions (Supplementary Table S6C). Rarefaction-specific distributions of global categorical PERMANOVA pseudo-F statistics, R^2^ values, and *p*-values are provided in Supplementary Table S6D.

### 3.4. Genus-Level Associations with Total MET-min/week

After prevalence filtering, 151 named genera were included in the exploratory Spearman analysis. Twenty-five genera showed nominal correlations with raw total MET-min/week at an unadjusted *p* < 0.05, including 19 positive and six negative correlations. However, none remained significant after Benjamini–Hochberg FDR correction across all 151 tested genera (Supplementary Table S9). In the compositional sensitivity analysis using ANCOM-BC2, 30 genera had FDR-adjusted q-values < 0.05 before application of the pseudo-count sensitivity criterion. None of these genera passed the pseudo-count sensitivity criterion. No genus was considered robustly associated with total MET-min/week (Supplementary Table S10).

## 4. Discussion

Previous studies have reported associations between physical activity and gut microbial diversity and community structure [19,20,21,22,23]. In this exploratory study, self-reported total MET-min/week was associated with the Shannon index and with variation in microbial community composition in young, apparently healthy Korean men. No significant differences in alpha diversity were observed among the PA categories, while categorical beta-diversity findings differed according to the distance metric. No robust genus-level association with total MET-min/week was identified.

Alpha diversity describes within-sample microbial diversity and may reflect microbial richness, evenness, or both, depending on the metric [34]. In the primary continuous analyses, total MET-min/week was positively associated with the Shannon index, whereas the associations with Observed features and Fisher’s alpha did not meet the FDR-adjusted significance threshold. Because the Shannon index incorporates both richness and the distribution of microbial abundances, this result may reflect a modest association with overall within-sample diversity rather than a clear increase in microbial richness [34]. The difference between the categorical and continuous findings may partly reflect the loss of information that can occur when a continuous exposure is divided into discrete groups [35,36]. In sensitivity analyses adjusted for age and BMI, the Shannon association remained significant, and the associations with Observed features and Fisher’s alpha also met the FDR-adjusted significance threshold. However, adjustment for age and BMI does not exclude residual confounding by diet or other unmeasured lifestyle factors.

Previous studies examining physical activity or cardiorespiratory fitness in relation to gut microbial alpha diversity have produced heterogeneous findings. Estaki et al. reported an association between objectively measured cardiorespiratory fitness and taxonomic richness in healthy adults [37], whereas a systematic review and meta-analysis found no consistent effect of physical activity on microbial alpha diversity across studies, although greater richness tended to be observed in athletes compared with non-athletes [3]. The differences observed among the alpha-diversity metrics in this study are consistent with this variability across previous studies. Given the modest sample size, reliance on self-reported PA, and limited control of potential lifestyle confounders, the Shannon association should be regarded as exploratory and requires confirmation using objective PA measurements and more comprehensive covariate assessment. Higher microbial diversity does not necessarily reflect a healthier gut microbiome, and its biological relevance depends on microbial function and host characteristics [38,39]. The biological relevance of the Shannon association in this young, apparently healthy population remains uncertain.

The categorical beta-diversity results differed according to the distance metric. Global differences among PA categories remained significant after FDR correction for Jaccard and unweighted UniFrac, whereas Bray–Curtis and Jensen–Shannon were not significant. Jaccard is based on taxon presence or absence, and unweighted UniFrac additionally incorporates phylogenetic relationships without weighting taxa by relative abundance. In contrast, Bray–Curtis and Jensen–Shannon incorporate abundance information [40,41]. These results showed that differences among PA categories were detected for metrics reflecting community membership and phylogenetic composition, but not for abundance-sensitive metrics. The findings should be interpreted cautiously because they varied according to the distance metric used. Global and pairwise PERMDISP analyses were not significant, indicating that the PERMANOVA results were not accompanied by significant differences in within-group dispersion. Jaccard and unweighted UniFrac remained FDR-significant in 71 of the 100 rarefaction-specific analyses, indicating some variability in the categorical findings across repeated rarefactions.

In the primary continuous analyses, total MET-min/week was associated with variation in microbial community composition across all four distance metrics after FDR correction. These models explained approximately 2.7–3.3% of the variation in community composition. The associations were also observed after adjustment for age and BMI and after log1p transformation of total MET-min/week. Across the 100 rarefaction-specific analyses, the raw and age- and BMI-adjusted models met the FDR-adjusted q < 0.05 threshold in all 100 iterations for all four metrics, whereas the log1p-transformed models met this threshold in 97–100 iterations. These repeated-rarefaction analyses indicate stability across repeated subsampling of the same participants and do not represent independent biological replications. Previous systematic reviews have reported considerable heterogeneity in exercise-related gut microbiome findings, with differences in exercise exposure, participant characteristics, dietary control, and study design contributing to variation among studies [22,23]. Exercise intensity and duration may also influence microbiome responses [42]. In this study, total MET-min/week combined walking, moderate-intensity, and vigorous-intensity activities into a single self-reported measure. Participants with similar total MET-min/week may have accumulated their activity through different combinations of intensity and duration. Although intensity-specific PA components were summarized descriptively, they were not analyzed as separate microbiome exposures. For this reason, the results should be interpreted in relation to total self-reported PA rather than a specific exercise intensity or training modality. Previous studies conducted in endurance-exercise settings have reported exercise-associated changes in specific taxa, including increases in *Veillonella* following prolonged endurance exercise [43,44]. However, these findings are not directly comparable with this study, in which habitual PA was assessed using self-reported total MET-min/week rather than a defined exercise intervention or endurance event. In the exploratory Spearman analysis, 25 genera showed nominal correlations with total MET-min/week, but none remained significant after FDR correction across the 151 tested genera. In the ANCOM-BC2 sensitivity analysis, 30 genera had FDR-adjusted q-values < 0.05, but none passed the pseudo-count sensitivity criterion. No robust genus-level association with total MET-min/week was identified. The genus-level results should be interpreted cautiously given multiple testing and the sensitivity of taxonomic analyses to analytical assumptions and confounding [42,45].

Exercise has been associated with changes in gut microbial composition and microbial function in human intervention studies [46]. Short-chain fatty acids (SCFA), including acetate, propionate, and butyrate, are microbial fermentation products involved in host metabolic and signaling processes [47]. PA-related changes in gut microbial composition and SCFA concentrations have also been reported in human studies [48]. However, this study assessed microbial taxonomy using 16S rRNA sequencing and did not measure fecal SCFA, microbial genes, transcripts, metabolites, intestinal permeability, or host immune and metabolic biomarkers. These data cannot determine whether the observed taxonomic associations were accompanied by changes in microbial metabolic function or host physiology.

This study has several limitations. First, PA was assessed using the IPAQ-SF, which is susceptible to measurement error and overreporting relative to objective PA assessment [49,50]. The C1–C3 categories were study-specific and data-derived and were neither prospectively specified nor equivalent to the canonical IPAQ Low, Moderate, and High classifications. Treating total MET-min/week as the primary continuous exposure did not eliminate measurement error in the underlying self-reported PA values. Walking, moderate-intensity PA, and vigorous-intensity PA were summarized descriptively but were not analyzed as separate microbiome exposures. In addition, a valid sitting-duration variable could not be derived. Future studies should incorporate objective PA measurements and more detailed assessment of activity intensity, duration, and sedentary behavior.

Second, validated dietary intake data were unavailable. Diet is an important determinant of gut microbial composition [13,14,15], but the dietary-preference item collected in this study did not quantify food, nutrient, fiber, or energy intake and could not be used for covariate adjustment. Traditional Korean dietary practices, including fermented-food consumption, may also influence gut microbial composition [29,30,31,32]. Smoking, habitual alcohol intake, sleep, stress, bowel habits, recent infections, probiotic or prebiotic use, and detailed training history were also not systematically quantified. Although age and BMI were included in sensitivity analyses, these adjustments did not account for diet or the other unmeasured lifestyle factors. Residual confounding by these factors cannot be excluded.

Third, the sample size was modest, the PA-category group sizes were unequal, and the study was not designed for extensive multivariable adjustment. Limited statistical precision may have reduced the ability to detect small associations, particularly after correction for multiple testing. The small effect sizes observed for the continuous beta-diversity associations also require confirmation in independent samples. Beta-diversity analyses were based on distance matrices averaged across 100 rarefactions. The statistical properties of PERMANOVA applied to these averaged matrices have not been formally established for all four distance metrics used in this study. Accordingly, the averaged-matrix results were interpreted together with the distributions of the 100 rarefaction-specific analyses when evaluating the stability of the findings.

Fourth, the cross-sectional design prevents inference regarding temporal direction or causality. It cannot be determined whether PA influenced the gut microbiota, whether pre-existing microbial characteristics were related to activity behavior, or whether both were influenced by unmeasured lifestyle or physiological factors. The restricted sample of young Korean men, most of whom were recruited from a university student population, also limits generalizability to women, older adults, clinical populations, and individuals from other geographic or cultural backgrounds.

Fifth, only the 131-bp forward portion of the V4 amplicon was used because the 2 × 150-bp sequencing configuration provided insufficient overlap for reliable paired-end reconstruction. Although the taxonomic classifier was trained to match the target region, read length, and orientation of the query sequences, the short 16S V4 fragment limited taxonomic resolution, particularly at the species level and potentially for some genera [51,52]. The genus-level findings should be interpreted conservatively. In addition, 16S rRNA relative abundance data are compositional and do not directly quantify absolute microbial abundance [53]. Absolute microbial abundance was not measured in this study.

Sixth, the time of day of stool collection, the exact interval between defecation and freezing, and acute strenuous exercise immediately before sample collection were not standardized or recorded. Variation in these pre-analytical factors may have contributed to between-participant microbiome differences [54]. Finally, the study did not include shotgun metagenomics, transcriptomics, metabolomics, fecal SCFA measurements, or detailed host phenotyping. Functional interpretations remain hypothesis-generating.

## 5. Conclusions

In summary, this exploratory cross-sectional analysis identified small associations of self-reported total MET-min/week with the Shannon index and gut microbial community composition, while categorical findings varied according to the diversity metric, and no robust genus-level association was identified. Because the primary analyses were largely unadjusted for lifestyle confounders, these findings require independent confirmation and should not be interpreted as evidence that greater PA modifies the gut microbiome. Longitudinal and controlled intervention studies combining objective PA and dietary assessments with metagenomic, metabolomic, and other functional analyses are needed to determine whether PA-related microbial differences are reproducible and biologically meaningful.

## Data Availability

The datasets generated and analyzed during the current study include human microbiome sequencing data. Due to participant privacy and Institutional Review Board restrictions, raw sequencing reads are not publicly available. De-identified summary data (ASV tables and diversity metrics) and analysis scripts are available from the corresponding author upon reasonable request; key summary-level results are provided in the Supplementary Materials.

## Supplementary Materials

The following supporting information can be downloaded at: https://www.mdpi.com/article/10.3390/sports14100436/s1, Table S1: Participant characteristics by physical activity category; Table S2: Sequencing and bioinformatic quality-control summary for the corrected QIIME 2 workflow; Table S3: Group-wise alpha-diversity values based on participant-level medians from 100 repeated rarefactions to 22,000 reads per sample; Table S4: Global and pairwise comparisons of alpha diversity across PA categories; Table S5: Primary and sensitivity analyses of associations between total MET-min/week and alpha diversity; Table S6: Global beta-diversity analyses across physical activity categories; Table S7: Pairwise beta-diversity comparisons among physical activity categories; Table S8: Continuous associations of total MET-min/week with beta diversity and sensitivity analyses; Table S9: Exploratory genus-level correlations with total MET-min/week after prevalence filtering; Table S10: ANCOM-BC2 compositional sensitivity analysis of genus-level associations with total MET-min/week.

## Abbreviations

The following abbreviations are used in this manuscript:

PA	Physical activity
WHO	World Health Organization
NGS	Next-generation sequencing
SCFA	Short-chain fatty acids
MET	Metabolic equivalent
IPAQ-SF	International Physical Activity Questionnaire-Short Form
ASVs	Amplicon sequence variants
DADA2	Divisive Amplicon Denoising Algorithm 2
QIIME 2	Quantitative Insights Into Microbial Ecology 2
FDR	False discovery rate
PERMANOVA	Permutational Multivariate Analysis of Variance
PERMDISP	Permutational Multivariate Analysis of Dispersion
PCoA	Principal coordinate analysis
BH	Benjamini–Hochberg
BCa	Bias-corrected and accelerated
