# Supplementary material for: Associations of Self-Reported Physical Activity with Gut Microbial Diversity and Community Structure in Young, Apparently Healthy Korean Men
Source: Sports (Basel). 2026 Oct 7;14(10):436. doi: 10.3390/sports14100436 (PMC13709621; doi:10.3390/sports14100436)
Supplement: Supplementary file 1 [file sports-14-00436-s001.zip › Supplementary_Tables_revised.pdf]

Supplementary Table S1. Participant characteristics by physical activity category

(A) Participant characteristics

	C1 (≤1500 MET-min/week)	C2 (1501-3000 MET-min/week)	C3 (≥3001 MET-min/week)	p-value
N	13	14	22	
Age, y	22.8 ± 2.7	22.4 ± 2.4	21.1 ± 1.4	0.124
Height, cm	173.7 ± 6.1	174.9 ± 6.3	176.0 ± 3.8	0.448
Weight, kg	69.5 ± 7.7	74.2 ± 8.5	73.7 ± 9.4	0.307
BMI, kg/m^2	23.0 ± 2.3	24.2 ± 2.5	23.8 ± 2.8	0.475
Total MET-min/week	753.8± 419.5	2346.5± 380.1	4564.5± 1008.1	<0.001

Values are mean ± SD for C1 (≤ 1500 MET-min/week), C2 (1501–3000 MET-min/week), and C3 (≥3001 MET-min/week). Group differences were tested by one-way ANOVA. As expected from the category definitions, total MET-min/week differed among the three PA categories ($p < 0.001$).

(B) Distribution of physical activity components

Physical activity variable	Overall (n = 49)	C1 (n = 13)	C2 (n = 14)	C3 (n = 22)
Walking, MET-min/week	693 [495–1386] (132–2772)	330 [297–495] (132–990)	577.5 [495–948.8] (132–1980)	1386 [693–1856.3] (495–2772)
Moderate-intensity PA, MET-min/week	480 [120–960] (0–2400)	80 [0–240] (0–720)	480 [150–660] (0–800)	1020 [510–1170] (0–2400)
Vigorous-intensity PA, MET-min/week	1440 [480–1920] (0–5040)	0 [0–320] (0–720)	1320 [960–1440] (0–2240)	2040 [1740–2880] (480–5040)
Total MET-min/week, median [IQR] (range)	2733 [1410–4266] (132–7026)	735 [330–1036] (132–1410)	2410 [2096.3–2655] (1620–2780)	4440 [3867.8–5013] (3230–7026)
Total MET-min/week, mean ± SD	2919.8 ± 1770.3	753.8 ± 419.5	2346.5 ± 380.1	4564.5 ± 1008.1

PA-component values in (B) are presented as median [Q1–Q3] (range), except for the additional mean ± SD summary of total MET-min/week. Walking, moderate-intensity, and vigorous-intensity MET-min/week were calculated using IPAQ-SF coefficients of 3.3, 4.0, and 8.0, respectively. C1, C2, and C3 were defined as ≤1500, 1501–3000, and ≥3001 total MET-min/week, respectively. The values in (B) are descriptive. Intensity-specific PA components

were not analyzed as separate microbiome exposures. A valid sitting-duration variable could not be derived because the response options for the sitting-related questionnaire item recorded frequency in days per week rather than sitting duration.

Supplementary Table S2. Sequencing and bioinformatic quality-control summary for the corrected QIIME 2 workflow

(A) Sequence-processing summary

Processing stage	Reads	Retention	ASVs	Additional information
Input forward reads	3,133,927	100.00%	-	Forward reads from 49 samples
After quality filtering	2,639,368	84.22% of input	-	DADA2 denoise-single
After denoising	2,608,196	83.22% of input	-	-
After chimera removal	2,549,665	81.36% of input	3,656	All representative sequences were 131 bp
Feature table used for diversity analyses	2,527,242	99.12% of non-chimeric reads	3,034	All ASVs were retained after SEPP placement

A total of 58,531 reads, corresponding to 2.24% of denoised reads, were removed as chimeric. Features not assigned to Bacteria and features classified as chloroplast or mitochondria were removed before diversity analysis. No ASVs or reads were excluded during SEPP phylogenetic placement.

(B) Paired-end merging feasibility

Processing stage	Read pairs	Additional information
Input	3,133,927	Full available forward and reverse read lengths
After filtering	2,409,847	76.90% of input
After denoising	2,363,232	75.41% of input
Merged	4,464	0.19% of denoised read pairs
Non-chimeric	4,424	99.10% of merged reads

(C) Pre-rarefaction library size by PA category

PA category	n	Median [Q1–Q3], reads	Range, reads
C1	13	39,339 [35,466–60,477]	22,766–72,182
C2	14	52,691 [42,971.8–59,238.8]	31,940–91,537
C3	22	55,560 [40,867.8–65,487.5]	23,384–92,453

(D) Sequencing-depth analyses

Analysis	Statistics	p-value
Library size across PA categories	Kruskal–Wallis H = 3.0523, df = 2	0.2174
Library size vs. total MET-min/week	Spearman's ρ = 0.1882	0.1952
Library size vs. unrarefied observed ASV count	Spearman's ρ = 0.4823	0.000449

Library size was calculated from the feature table before rarefaction. C1, C2, and C3 were defined as ≤1500, 1501–3000, and ≥3001 MET-min/week, respectively. Sequencing-depth analyses were treated as quality-control analyses and are reported using raw two-sided p-values.

Supplementary Table S3. Group-wise alpha-diversity values based on participant-level medians from 100 repeated rarefactions to 22,000 reads per sample

Measure	Group	n	Mean ± SD	Median [Q1–Q3]
Observed features	C1	13	166.73 ± 58.94	148.00 [131.00–210.50]
	C2	14	200.25 ± 64.82	193.00 [173.00–214.50]
	C3	22	209.25 ± 64.14	188.25 [156.25–264.63]
Shannon	C1	13	5.05 ± 0.69	5.11 [4.57–5.38]
	C2	14	5.36 ± 0.67	5.44 [4.87–5.61]
	C3	22	5.56 ± 0.57	5.58 [5.17–5.92]
Fisher's alpha	C1	13	24.85 ± 10.28	21.33 [18.50–32.25]
	C2	14	30.77 ± 11.87	29.12 [25.60–32.98]
	C3	22	32.38 ± 11.84	28.27 [22.72–42.33]

Values are presented as the mean ± standard deviation and median [first quartile–third quartile] of the participant-level summary values. For each participant, Observed features, the Shannon index, and Fisher's alpha were calculated in 100 repeated rarefactions to 22,000 reads per sample, and the median across the 100 rarefaction-specific values was used as the participant-level summary. C1, C2, and C3 were defined as ≤1500, 1501–3000, and ≥3001 MET-min/week, respectively.

Supplementary Table S4. Global and pairwise comparisons of alpha diversity across PA categories

(A) Global comparisons

Measure	Kruskal–Wallis χ^2	df	ε^2	p-value	FDR-adjusted q-value
Observed features	4.207	2	0.048	0.1220	0.1220
Shannon index	5.588	2	0.078	0.0612	0.1220
Fisher's alpha	4.207	2	0.048	0.1220	0.1220

(B) Pairwise comparisons

Measure	Comparison	Wilcoxon W	Raw p-value	FDR-adjusted q-value
Observed features	C1 vs. C2	64.5	0.2069	0.3104
	C1 vs. C3	79.5	0.0314	0.0943
	C2 vs. C3	153.0	0.9871	0.9871
Shannon index	C1 vs. C2	65.0	0.2159	0.3239
	C1 vs. C3	74.0	0.0194	0.0581
	C2 vs. C3	124.0	0.3384	0.3384
Fisher's alpha	C1 vs. C2	64.5	0.2069	0.3104
	C1 vs. C3	79.5	0.0314	0.0943
	C2 vs. C3	153.0	0.9871	0.9871

Global differences were assessed using Kruskal-Wallis tests. Pairwise comparisons were conducted using two-sided Wilcoxon rank-sum tests. Benjamini-Hochberg correction was applied across the three global alpha-diversity tests and separately across the three pairwise comparisons within each alpha-diversity metric.

Supplementary Table S5. Primary and sensitivity analyses of associations between total MET-min/week and alpha diversity

(A) Primary unadjusted associations between total MET-min/week and participant-level median alpha-diversity values

Metric	Spearman's ρ	95% bootstrap CI	p-value	FDR-adjusted q-value
Observed features	0.2803	−0.0331 to 0.5212	0.0511	0.0511
Shannon index	0.3567	0.0821 to 0.5812	0.0119	0.0356
Fisher's alpha	0.2803	−0.0242 to 0.5249	0.0511	0.0511

(B) Age- and BMI-adjusted sensitivity analyses of associations between total MET-min/week and alpha diversity

Metric	Partial Spearman's ρ	p-value	FDR-adjusted q-value
Observed features	0.2945	0.0445	0.0445
Shannon index	0.3697	0.0105	0.0316
Fisher's alpha	0.2945	0.0445	0.0445

(C) Stability of categorical and continuous alpha-diversity results across 100 rarefaction iterations

Metric	Global PA-category test with q < 0.05, n/100	Continuous MET correlation with q < 0.05, n/100
Observed features	0/100	29/100
Shannon index	0/100	100/100
Fisher's alpha	0/100	29/100

(A) presents Spearman rank correlations between raw total MET-min/week and participant-level median alpha-diversity values from 100 repeated rarefactions to 22,000 reads per sample. The 95% bootstrap CIs for Spearman's ρ in (A) were estimated from 10,000 participant-level resamples using the bias-corrected and accelerated (BCa) method. (B) presents partial Spearman rank correlations for the same associations after adjustment for age and BMI. Benjamini–Hochberg FDR correction was applied separately across the three alpha-diversity metrics for (A) and (B). (C) shows the number of rarefaction iterations in which the unadjusted global PA-category test or continuous MET correlation met the FDR-adjusted $q < 0.05$ threshold.

Supplementary Table S6. Global beta-diversity analyses across physical activity categories

(A) Global PERMANOVA across PA categories

Distance metric	Pseudo-F	R^2	p-value	FDR-adjusted q-value
Jaccard	1.2206	0.0504	0.0191	0.0432
Bray–Curtis	1.1794	0.0488	0.1471	0.1783
Jensen–Shannon	1.1169	0.0463	0.1783	0.1783
Unweighted UniFrac	1.2324	0.0509	0.0216	0.0432

(B) Global PERMDISP across PA categories

Distance metric	F	p-value	FDR-adjusted q-value
Jaccard	1.1603	0.3200	0.3200
Bray–Curtis	2.2464	0.1188	0.2376
Jensen–Shannon	3.1556	0.0569	0.2276
Unweighted UniFrac	1.3823	0.2644	0.3200

(C) Stability across 100 repeated rarefactions

Distance metric	PERMANOVA $p < 0.05$, n/100	PERMANOVA $q < 0.05$, n/100	PERMDISP $p < 0.05$, n/100	PERMDISP $q < 0.05$, n/100
Jaccard	100/100	71/100	0/100	0/100
Bray–Curtis	0/100	0/1 00	0/100	0/100
Jensen–Shannon	0/100	0/100	29/100	0/100
Unweighted UniFrac	100/100	71/100	0/100	0/100

(D) Distribution of global PERMANOVA results across 100 repeated rarefactions

Distance metric	Pseudo-F, median [Q1–Q3]	R^2, median [Q1–Q3]	p-value, median [Q1–Q3]	p-value range (min-max)
Jaccard	1.2217 [1.2148–1.2263]	0.0504 [0.0502–0.0506]	0.0200 [0.0182–0.0220]	0.0148–0.0291
Bray–Curtis	1.1790 [1.1756–1.1831]	0.0488 [0.0486–0.0489]	0.1538 [0.1468–0.1584]	0.1256–0.1727
Jensen–Shannon	1.1167 [1.1146–1.1189]	0.0463 [0.0462–0.0464]	0.1767 [0.1708–0.1810]	0.1567–0.1878
Unweighted UniFrac	1.2282 [1.2147–1.2522]	0.0507 [0.0502–0.0516]	0.0211 [0.0153–0.0267]	0.0085–0.0433

Global differences among PA categories were assessed using PERMANOVA, and homogeneity of multivariate dispersion was assessed using PERMDISP, with 9,999 permutations. For each beta-diversity metric, distance matrices obtained from 100 repeated rarefactions to 22,000 reads per sample

were averaged element-wise, and the resulting averaged distance matrix was used for the global categorical analyses in (A) and (B). Benjamini–Hochberg correction was applied across the four distance metrics separately for PERMANOVA and PERMDISP. (C) shows the numbers of rarefaction-specific analyses meeting the raw $p < 0.05$ and FDR-adjusted $q < 0.05$ thresholds. (D) summarizes the distributions of rarefaction-specific PERMANOVA pseudo-F statistics, R^2 values, and raw p-values across the 100 repeated rarefactions. Values in (D) are presented as median [Q1–Q3], and the p-value range represents the minimum and maximum raw PERMANOVA p-values. The repeated-rarefaction analyses were used to assess the stability of the results across repeated rarefactions of the same participants and do not represent independent biological replications.

Supplementary Table S7. Pairwise beta-diversity comparisons among physical activity categories

(A) Pairwise PERMANOVA

Distance metric	Comparison	Pseudo-F	R^2	p-value	BH-adjusted q-value
Jaccard	C1 vs. C2	1.1031	0.0423	0.1588	0.2382
	C1 vs. C3	1.5660	0.0453	0.0010	0.0030
	C2 vs. C3	0.9722	0.0278	0.5209	0.5209
Bray–Curtis	C1 vs. C2	1.0984	0.0421	0.3132	0.4698
	C1 vs. C3	1.6106	0.0465	0.0168	0.0504
	C2 vs. C3	0.7831	0.0225	0.7954	0.7954
Jensen–Shannon	C1 vs. C2	1.1275	0.0432	0.2109	0.3164
	C1 vs. C3	1.4310	0.0416	0.0186	0.0558
	C2 vs. C3	0.7769	0.0223	0.9075	0.9075
Unweighted UniFrac	C1 vs. C2	1.0491	0.0403	0.2675	0.2675
	C1 vs. C3	1.3405	0.0390	0.0242	0.0569
	C2 vs. C3	1.2728	0.0361	0.0379	0.0569

(B) Pairwise PERMDISP

Distance metric	Comparison	F	p-value	BH-adjusted q-value
Jaccard	C1 vs. C2	0.7805	0.3926	0.4985
	C1 vs. C3	2.0948	0.1537	0.4611
	C2 vs. C3	0.4701	0.4985	0.4985
Bray–Curtis	C1 vs. C2	3.7733	0.0622	0.1866
	C1 vs. C3	1.7978	0.1880	0.2820
	C2 vs. C3	1.0783	0.3016	0.3016
Jensen–Shannon	C1 vs. C2	5.7832	0.0225	0.0675
	C1 vs. C3	2.4969	0.1194	0.1791
	C2 vs. C3	1.4083	0.2435	0.2435
Unweighted UniFrac	C1 vs. C2	0.0108	0.9205	0.9205
	C1 vs. C3	1.5755	0.2230	0.3345
	C2 vs. C3	1.7641	0.1926	0.3345

Pairwise differences among PA categories were assessed using PERMANOVA, and pairwise homogeneity of multivariate dispersion was assessed using PERMDISP, with 9,999 permutations. For each beta-diversity metric, distance matrices obtained from 100 repeated rarefactions to 22,000 reads per sample were averaged, and pairwise analyses were performed using the resulting averaged distance matrix. Benjamini–Hochberg correction was applied separately within each distance metric across the three pairwise comparisons (C1 vs. C2, C1 vs. C3, and C2 vs. C3) for PERMANOVA and PERMDISP. Statistical significance was defined as an FDR-adjusted q-value < 0.05.

Supplementary Table S8. Continuous associations of total MET-min/week with beta diversity and sensitivity analyses

(A) Continuous PERMANOVA using standardized raw total MET-min/week

Distance metric	Pseudo-F	R^2	p-value	FDR-adjusted q-value
Jaccard	1.4051	0.0290	0.0080	0.0320
Bray–Curtis	1.6001	0.0329	0.0226	0.0328
Jensen–Shannon	1.3781	0.0285	0.0378	0.0378
Unweighted UniFrac	1.3148	0.0272	0.0246	0.0328

(B) Continuous PERMANOVA using standardized raw total MET-min/week adjusted for age and BMI

Distance metric	Pseudo-F	R^2	p-value	FDR-adjusted q-value
Jaccard	1.4797	0.0305	0.0050	0.0146
Bray–Curtis	1.7137	0.0353	0.0099	0.0146
Jensen–Shannon	1.4841	0.0307	0.0146	0.0146
Unweighted UniFrac	1.3945	0.0288	0.0124	0.0146

(C) Continuous PERMANOVA using standardized log1p-transformed total MET-min/week

Distance metric	Pseudo-F	R^2	p-value	FDR-adjusted q-value
Jaccard	1.5471	0.0319	0.0018	0.0036
Bray–Curtis	1.9412	0.0397	0.0017	0.0036
Jensen–Shannon	1.6771	0.0345	0.0032	0.0043
Unweighted UniFrac	1.2894	0.0267	0.0320	0.0320

(D) Stability across 100 repeated rarefactions

Distance metric	Raw MET q < 0.05, n/100	Age- and BMI-adjusted raw MET q<0.05, n/100	Log1p MET q<0.05, n/100
Jaccard	100/100	100/100	100/100
Bray–Curtis	100/100	100/100	100/100
Jensen–Shannon	100/100	100/100	100/100
Unweighted UniFrac	100/100	100/100	97/100

(E) Distribution of continuous PERMANOVA results across 100 repeated rarefactions

Model	Distance metric	Pseudo-F, median [Q1–Q3]	R^2, median [Q1–Q3]	p-value, median [Q1–Q3]	p-value range (min-max)
Raw MET	Jaccard	1.4050 [1.3983–1.4125]	0.0290 [0.0289–0.0292]	0.0088 [0.0082–0.0098]	0.0064–0.0130
	Bray–Curtis	1.6009 [1.5923–1.6066]	0.0329 [0.0328–0.0331]	0.0222 [0.0210–0.0233]	0.0182–0.0257
	Jensen–Shannon	1.3780 [1.3744–1.3816]	0.0285 [0.0284–0.0286]	0.0360 [0.0343–0.0373]	0.0298–0.0411
	Unweighted UniFrac	1.3154 [1.2995–1.3292]	0.0272 [0.0269–0.0275]	0.0264 [0.0221–0.0305]	0.0126–0.0454
Age/BMI-adjusted	Jaccard	1.4801 [1.4732–1.4870]	0.0306 [0.0304–0.0307]	0.0041 [0.0037–0.0047]	0.0023–0.0066
	Bray–Curtis	1.7141 [1.7062–1.7207]	0.0353 [0.0352–0.0354]	0.0112 [0.0103–0.0121]	0.0087–0.0145
	Jensen–Shannon	1.4843 [1.4808–1.4876]	0.0307 [0.0306–0.0307]	0.0152 [0.0145–0.0160]	0.0123–0.0188
	Unweighted UniFrac	1.3932 [1.3775–1.4129]	0.0288 [0.0284–0.0292]	0.0124 [0.0103–0.0145]	0.0059–0.0232
Log1p MET	Jaccard	1.5482 [1.5419–1.5530]	0.0319 [0.0318–0.0320]	0.0022 [0.0018–0.0025]	0.0009–0.0035
	Bray–Curtis	1.9412 [1.9353–1.9472]	0.0397 [0.0395–0.0398]	0.0026 [0.0023–0.0030]	0.0013–0.0044
	Jensen–Shannon	1.6769 [1.6731–1.6803]	0.0344 [0.0344–0.0345]	0.0032 [0.0028–0.0036]	0.0016–0.0049
	Unweighted UniFrac	1.2900 [1.2754–1.3019]	0.0267 [0.0264–0.0270]	0.0331 [0.0282–0.0375]	0.0170–0.0535

Raw and log1p-transformed total MET-min/week were standardized before analysis. For each beta-diversity metric, distance matrices obtained from 100 repeated rarefactions to 22,000 reads per sample were averaged element-wise, and the resulting averaged distance matrix was used for the PERMANOVA analyses in (A)–(C). (A) shows the primary models using standardized raw total MET-min/week. (B) shows the age- and BMI-adjusted sensitivity analyses, with the marginal effect of total MET-min/week reported. (C) shows the sensitivity analyses using standardized log1p-transformed total MET-min/week. PERMANOVA was performed using 9,999 permutations, with Benjamini–Hochberg correction applied across the four distance metrics separately within each model. (D) shows the number of rarefaction-specific analyses meeting the FDR-adjusted $q < 0.05$ threshold. (E) summarizes the distributions of rarefaction-specific PERMANOVA pseudo-F statistics, R^2 values, and raw p-values across the 100 repeated rarefactions for the raw, age- and BMI-adjusted, and log1p-transformed models. Values in (E) are presented as median [Q1–Q3], and the p-value range represents the minimum and

maximum raw PERMANOVA p-values. The repeated-rarefaction analyses were used to assess the stability of the results across repeated rarefactions of the same participants and do not represent independent biological replications.

Supplementary Table S9. Exploratory genus-level correlations with total MET-min/week after prevalence filtering

(A) Positive nominal correlations with total MET-min/week

Genus	Spearman's ρ	Raw p-value	FDR-adjusted q-value	Prevalence n (%)	Mean relative abundance (%)
Negativibacillus	0.4163	0.0029	0.2132	27 (55.1)	0.0568
Adlercreutzia	0.3895	0.0057	0.2132	26 (53.1)	0.0960
Dorea	0.3849	0.0063	0.2132	45 (91.8)	1.5443
Romboutsia	0.3833	0.0066	0.2132	46 (93.9)	0.7839
Lachnospiraceae_UCG-008	0.3735	0.0082	0.2132	20 (40.8)	0.0269
Christensenellaceae_R-7_group	0.3664	0.0096	0.2132	26 (53.1)	0.2354
Colidextribacter	0.3652	0.0099	0.2132	29 (59.2)	0.0927
UCG-005	0.3505	0.0135	0.2396	24 (49.0)	0.1509
Fenollaria	0.3468	0.0146	0.2396	6 (12.2)	0.0014
Holdemania	0.3401	0.0168	0.2396	28 (57.1)	0.0126
Shuttleworthia	0.3352	0.0186	0.2396	11 (22.4)	0.0176
UCG-009	0.3339	0.0190	0.2396	16 (32.7)	0.0133
[Eubacterium]_hallii_group	0.3177	0.0261	0.2468	43 (87.8)	1.6893
Porphyromonas	0.3114	0.0294	0.2468	5 (10.2)	0.0008
Lachnospiraceae_NK4A136_group	0.3061	0.0324	0.2499	41 (83.7)	0.2821
NK4A214_group	0.3023	0.0348	0.2499	23 (46.9)	0.0957
[Eubacterium]_coprostanoligenes_group	0.2935	0.0407	0.2793	28 (57.1)	0.4507
UCG-010	0.2853	0.0469	0.2881	11 (22.4)	0.0607
[Eubacterium]_brachy_group	0.2843	0.0477	0.2881	13 (26.5)	0.0104

(B) Negative nominal correlations with total MET-min/week

Genus	Spearman's ρ	Raw p-value	FDR-adjusted q-value	Prevalence n (%)	Mean relative abundance (%)
Lactobacillus	−0.3259	0.0223	0.2468	37 (75.5)	0.5856
[Clostridium]_innocuum_group	−0.3173	0.0263	0.2468	34 (69.4)	0.0566
Gemella	−0.3151	0.0274	0.2468	23 (46.9)	0.0112
Fusobacterium	−0.3140	0.0280	0.2468	18 (36.7)	0.0800
Veillonella	−0.3024	0.0347	0.2499	42 (85.7)	0.5876

Eisenbergiella	−0.2863	0.0461	0.2881	9 (18.4)	0.0050

Spearman rank correlations were calculated between raw total MET-min/week and genus-level relative abundances. Relative abundances were calculated using the complete retained bacterial community within each participant as the denominator before genus-level filtering. Named genera detected in ≥10% of participants (≥5 of 49) were retained, resulting in 151 genera for hypothesis testing. Benjamini–Hochberg correction was applied across all 151 tested genera. The table shows genera with an unadjusted $p < 0.05$. None remained significant at $q < 0.05$. Prevalence indicates the number and percentage of participants with a non-zero abundance. Mean relative abundance is expressed as a percentage of the complete retained bacterial community.

Supplementary Table S10. ANCOM-BC2 compositional sensitivity analysis of genus-level associations with total MET-min/week

Genus	LFC	SE	W	Raw p	q
Weissella	1.0150	0.1074	9.4512	1.04×10^{-7}	1.58×10^{-5}
Family_XIII_AD3011_group	0.5481	0.1000	5.4839	1.65×10^{-5}	0.0012
Alloprevotella	1.5760	0.1136	13.8672	3.50×10^{-5}	0.0013
Butyricimonas	−0.6678	0.1134	−5.8892	3.94×10^{-5}	0.0013
Clostridia_UCG-014	1.1147	0.1613	6.9105	4.14×10^{-5}	0.0013
CAG-352	1.1967	0.1299	9.2140	0.0003	0.0064
Mogibacterium	0.7627	0.0980	7.7818	0.0006	0.0111
Eisenbergiella	−0.5072	0.0857	−5.9170	0.0006	0.0111
Lachnospiraceae_UCG-009	1.2167	0.0823	14.7921	0.0007	0.0112
Eubacterium	−0.5068	0.1038	−4.8824	0.0009	0.0131
Fenollaria	0.7132	0.0844	8.4481	0.0011	0.0134
[Eubacterium]_xylanophilum_group	0.6160	0.1175	5.2437	0.0012	0.0134
UCG-005	0.5896	0.1593	3.7019	0.0012	0.0134
Fournierella	−0.3765	0.0909	−4.1419	0.0014	0.0134
Pediococcus	−1.0691	0.0925	−11.5541	0.0014	0.0134
Solobacterium	−0.6826	0.1228	−5.5592	0.0014	0.0134
Negativibacillus	0.4903	0.1376	3.5627	0.0015	0.0134
Senegalimassilia	0.8111	0.0829	9.7879	0.0023	0.0190
[Clostridium]_innocuum_group	−0.6064	0.1876	−3.2332	0.0028	0.0226
[Eubacterium]_brachy_group	0.3526	0.0932	3.7831	0.0030	0.0226
[Ruminococcus]_gnavus_group	−0.7136	0.2262	−3.1544	0.0031	0.0226
[Eubacterium]_nodatum_group	0.5564	0.0889	6.2562	0.0033	0.0228
[Eubacterium]_ruminantium_group	0.4045	0.0888	4.5534	0.0039	0.0255
Lactococcus	−0.5024	0.1477	−3.4023	0.0047	0.0297
Moryella	0.3036	0.0863	3.5171	0.0065	0.0395
Catenibacillus	0.4133	0.0834	4.9588	0.0077	0.0448
UCG-002	0.4119	0.1452	2.8378	0.0082	0.0453
Megasphaera	−0.5540	0.1526	−3.6298	0.0084	0.0453
Raoultibacter	0.5039	0.0836	6.0300	0.0091	0.0476
Oxalobacter	−0.3094	0.0830	−3.7284	0.0098	0.0491

ANCOM-BC2 was used as a compositional sensitivity analysis with standardized total MET-min/week as the continuous explanatory variable. Raw genus-level counts were analyzed after applying a prevalence threshold of ≥10% of participants, resulting in 151 genera. Benjamini–Hochberg correction was applied across all 151 genera, and pseudo-count sensitivity analysis was enabled. The table presents the 30 genera with an FDR-adjusted q-value < 0.05 before application of the pseudo-count sensitivity criterion. None of these genera passed the pseudo-count sensitivity criterion. No genus was considered robustly associated with total MET-min/week. LFC represents the estimated log-fold change per 1-SD increase in total MET-min/week.
